# Transcutaneous vagus nerve stimulation as a pain modulator in knee osteoarthritis: a randomized controlled clinical trial

**DOI:** 10.1186/s12891-025-08288-6

**Published:** 2025-01-20

**Authors:** Gehad Gamal Elsehrawy, Maha Emad Ibrahim, Nermeen Hassan A.moneim, Mohamed Ahmed Hefny, Nashwa Kamel El Shaarawy

**Affiliations:** Department of Physical Medicine, Rheumatology and Rehabilitation, faculty of medicine, Suez Canal University, Ismailia, 41522 Egypt

**Keywords:** Osteoarthritis, Transcutaneous vagus nerve stimulation, Central sensitization, Functional outcomes

## Abstract

**Background:**

Our understanding of osteoarthritis (OA) has evolved from a degenerative disease to one in which low-grade, chronic inflammation plays a central role. In addition, evidence suggests that OA is accompanied by both peripheral and central nervous system sensitization that can cause pain. It has been demonstrated that transcutaneous vagus nerve stimulation (tVNS) can relieve pain, inflammation, and central sensitization in other conditions including fibromyalgia, pelvic pain, and headaches. We aimed to assess the efficacy and safety of tVNS on nociceptive pain, central sensitization, and physical function in knee OA.

**Methods:**

In this 12-week study, we stimulated the auricular branch of the vagus nerve with an auricular electrode connected to a transcutaneous electrical nerve stimulation device once a day for 3 days each week for 12 weeks. A total of 68 patients with chronic knee OA were randomly assigned to the active and sham groups (34 patients in each group). We used a variety of outcome measures, including the visual analog scale (VAS), pressure pain threshold (PPT), knee injury and osteoarthritis outcome score (KOOS), PainDETECT (PD-Q) and Douleur Neuropathique 4 (DN4) questionnaires. Outcome measures were recorded at baseline, At the end of the stimulation period, and then after 4 weeks.

**Results:**

In the active group, compared to baseline, there was a significant improvement in VAS scores between the first and second follow-up visits (*P* < 0.001). A significant improvement in PPT was seen in the right knee, left knee, and right elbow in active tVNS, and this improvement persisted for four weeks post-intervention. Meanwhile, in the sham group, right knee PPT was improved but not maintained. There were statistically significant improvements in the PD-Q and DN4 scores in the active tVNS group (*P* < 0.001), whereas in the sham group, DN4 questionnaire did not show any improvement. In terms of functional outcomes, the improvement in KOOS was significant only in the active group (31.44 ± 18.49, *P* < 0.001). No serious adverse events were observed.

**Conclusion:**

There is preliminary evidence to support the benefits of tVNS in OA, suggesting that neuromodulation can be used as an adjunct to existing pharmacological and non-pharmacological treatments.

**Trial registration:**

The study was registered on ClinicalTrials.gov (NCT05387135) on 24/05/2022.

**Supplementary Information:**

The online version contains supplementary material available at 10.1186/s12891-025-08288-6.

## Introduction

Osteoarthritis (OA) is the most common joint disorder in elderly individuals. The prevalence of symptomatic knee OA in two UK studies ranged from 11 to 19%, and estimates of 5–15% were noted in surveys undertaken in other countries [1]. As chronic pain and loss of function are common symptoms of OA, the socioeconomic burden is substantial. This burden costs in developed countries between 1.0% and 2.5% of gross domestic product [2]. Currently, no cure can prevent joint destruction. The Global Burden of Disease ranks OA of the hip and knee 11th in terms of its contribution to worldwide disability [3].

Over the past decade, we have seen a fundamental shift in our understanding of the mechanisms that cause pain in osteoarthritis, in which inflammation and central sensitization play an essential role. As a result, targeting pain sensitization with duloxetine [4] and vortioxetine [5] is considered crucial to improve pain management in OA. In addition, using transcranial direct current stimulation has demonstrated promising results in knee OA [6]. We are no longer viewing OA as just a degenerative disease but rather as a multifactorial disorder in which chronic low-grade inflammation plays a central role [7]. Old age, prior joint injuries, and obesity are all risk factors for joint damage, which triggers an immune response leading to chronic, low-grade inflammation and OA development [8].

Inflammation in OA is mediated by damage-associated molecular patterns, Toll-like receptors, the complement system, macrophages, and mast cells [9]. Cytokines, interleukin-1 (IL-1β), tumor necrosis factor (TNF), chemokines, growth factors, prostaglandins, leukotrienes, and neuropeptides are also included in the inflammatory process associated with OA [8]. In addition, prolonged activation of dorsal root ganglia by inflammatory cytokines originating in the joint may impact central nervous system pain pathways, particularly those controlling descending inhibitions, and result in central sensitization in chronic pain conditions such as OA. As a result, evidence points to both peripheral and central nervous system sensitization as sources of pain in OA [10, 11].

Interestingly, it was found that stimulation of the afferent vagus nerve may activate the hypothalamic-pituitary-adrenal axis, resulting in an anti-inflammatory effect [12]. A disruption of this anti-inflammatory vagal reflex has been found in various autoimmune and inflammatory disorders, including rheumatoid arthritis (RA), pancreatitis [13], inflammatory bowel disease, and other conditions in which inflammation plays a role in the pathogenesis [14].

Moreover, pharmacological activation of nicotinic acetylcholine receptors (a7nAChR) or electrical stimulation of the cholinergic anti-inflammatory pathway by vagus nerve stimulation (VNS) may lower cytokine production, delay joint destruction, and ameliorate clinical signs of arthritis [15]. This cholinergic anti-inflammatory reflex can also be stimulated either by electrical VNS with an implantable device close to the left cervical vagus nerve or by transcutaneous vagus nerve stimulation (tVNS), stimulating the auricular branch of the vagus nerve [16]. Furthermore, a recent exploratory small study suggested that VNS significantly reduced TNF and IL-6 production and lowered the severity of rheumatoid arthritis, even in some individuals with therapy-resistant illness [17]. Therapy with nicotine reduced TNF production in the colon and improved colitis, but subdiaphragmatic vagotomy of the ventral and dorsal vagus nerves raised the colitis disease activity score and markedly elevated TNF, IL-6, and IL-1b production in colon tissue [18].

Additionally, VNS is generally well tolerated and has been used to treat medication-resistant epilepsy in more than 100,000 individuals [17]. With few adverse effects during electrical stimulation of the nerve, such as hoarseness, dysphonia, and coughing. It is a safe procedure and well tolerated by patients [19]. VNS is currently approved as a treatment for epilepsy, depression, and tinnitus, while it is also being researched as a potential novel treatment for stroke, RA, Crohn’s disease, and heart failure [20].

Recent human and animal research shows mounting evidence that VNS can have potent analgesic benefits in addition to its anti-inflammatory effects. Inhibiting spinal nociceptive reflexes through stimulation of the vagal afferents may be beneficial for treating a variety of chronic pain syndromes, such as fibromyalgia, pelvic pain, and migraine [21, 22].

Considering tVNS’s efficacy on pain, inflammation, and central sensitization as well as its safety profile, we hypothesized that tVNS could be a new additional treatment for knee OA. This first proof-of-concept trial aimed to assess the safety and efficacy of tVNS on nociceptive pain, neuropathic pain, central sensitization, and physical function in individuals with knee OA.

## Materials and methods

### Study design and setting

We performed a single-blind, sham-controlled, randomized clinical trial to assess the use of transcutaneous VNS as a pain modulator in patients with knee OA. It was a single-center trial conducted at the Physical Medicine, Rheumatology, and Rehabilitation outpatient clinics at Suez Canal University Hospitals in Ismailia city, Egypt, between December 2019 and March 2022.

The study protocol was approved by the Committee of Ethical Research, Faculty of Medicine, Suez Canal University, (date of approval: 25/3/2019, Number 3905#). The study was conducted according to the Declaration of Helsinki and its subsequent modifications (193), and all patients provided written informed consent. The study was pre-registered on ClinicalTrials.gov (NCT05387135) on 24/05/2022.

### Participants

Patients over 18 years old with bilateral knee OA according to the American College of Rheumatology’s (ACR) classification criteria [23], and had pain intensity of at least 4 on the visual analogue scale (VAS) were eligible for inclusion. Prior to group allocation, baseline patients’ data was gathered. Individuals were excluded if they had any neurologic and musculoskeletal disorders affecting lower extremity function, total knee arthroplasty, arthrodesis, autoimmune disease, psychiatric disorders, or pregnancy. Also, patients who had cellulitis or skin ulceration at the area of therapy application, implanted electrical devices, or on selective serotonin and norepinephrine-reuptake inhibitor drugs and anti-convulsant drugs were excluded.

To allow for washout, non-steroidal anti-inflammatory drugs (NSAIDs) and other painkillers were discontinued for two weeks [24].

#### Experimental design

After that, research staff who had no further contact with the subjects randomly divided them into two groups: active and sham. Group assignments were prepared using a computer-generated random sequence and placed in sequentially numbered, sealed envelopes. Envelopes were opened only after the participant was enrolled.

The duration of the tVNS treatment in this study was three months, and the number of stimulation sessions was decided upon following previous research that demonstrated the effectiveness of tVNS in treating various chronic pain conditions [25]. Patients were blinded to group allocation, whereas the treating physician, who set the tVNS according to the protocol, was aware of the stimulation condition.

#### Active treatment

**Fig. 1:**
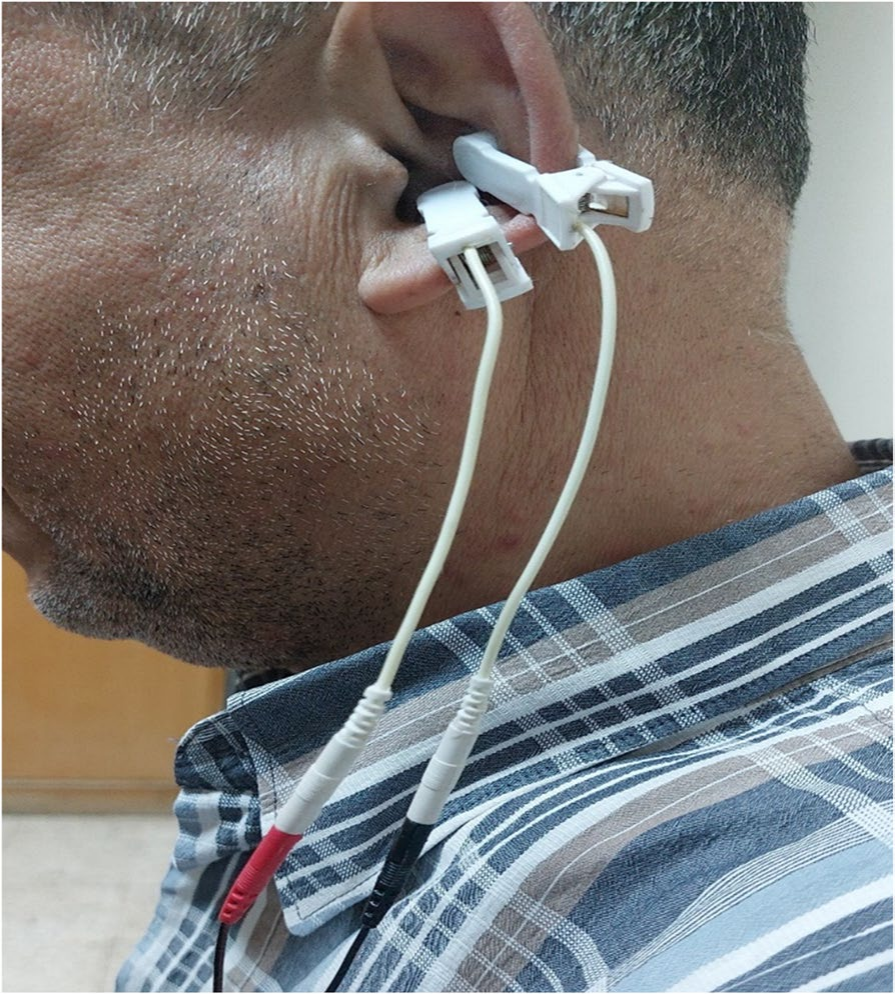
Active tVNS stimulation of a case in our study

A tVNS device (TENS 7000TM) made by Roscoe Medical Inc. was used to stimulate the afferents of the auricular branch of the vagus nerve (ABVN). The TENS 7000TM device, which consists of a stimulator unit and a bipolar stimulation electrode, is described as a nerve stimulator and a low-risk medical device (Instruction manual for TENS 7000). After being cleaned with an alcohol swab, the electrode was applied directly to the skin in the left cymba concha as shown in Fig. 1.

Before the experimental methods, subjects were accustomed to the stimulus for five minutes. The intensity was then gradually increased until the ideal intensity was obtained (i.e., clear tingling sensation, but not painful). Following that, patients are free to sit up or lie on their side. Due to habituation, the intensity was readjusted throughout the 30 min of continuous stimulation, aiming at the initial perceptive experience [26]. The stimulation for both groups lasted for 30 min once a day for 3 days per week for 12 weeks. The amplitude of the output current was between 0.25 and 2.0 mA as tolerated, with a 250 µs width at 25 Hz [17].

#### Sham procedure

For sham-t-VNS, stimulating electrodes were placed on the outer earlobe, which does not contain fibers of the ABVN. Patients were unaware of which site provides active stimulation to achieve blinding of treatment allocation. A protocol similar to that used for active stimulation was used [27].

All participants were initially evaluated before and after intervention and 4 weeks after the end of the sessions by full medical history, general and musculoskeletal examination, and the following outcome measures:

#### Outcome measures

1. Visual analog scale (VAS).
2. Quantitative sensory testing (QST): Pressure pain threshold (PPT).
3. The central sensitization inventory (CSI).
4. Knee injury and Osteoarthritis Outcome Score (KOOS).
5. Physical function tests: Performance-based chair stand test (CST) and timed up and go test.
6. PainDETECT questionnaire.
7. Douleur neuropathique 4 (DN4) questionnaire.
8. Hospital Anxiety Depression Scale (HADS).
9. Kellgren-Lawrence (KL) Radiological classification.

1. Visual analog scale (VAS):
  On a line measuring one centimeter and graded from 0 to 10, patients were asked to indicate the degree of their knee pain, with “0” denoting no pain and “10” denoting the greatest pain they could imagine. It is also possible to categorize pain as none, mild, moderate, or severe (none = 0, mild = 1–4, moderate = 5–6, and severe = 7–10) [28].
2. Central sensitization:
  - Pressure pain threshold (PPT):
    Tests of PPT are performed at a diseased site to measure local pain sensitivity as a surrogate for peripheral and/or central sensitization. Tests performed at distant, nonpainful sites test widespread hypersensitivity, a sign of central sensitization [29]. Using standardized instructions, we applied the pressure algometer to the following sites: both knees (medial joint line) as well as the right elbow over the extensor carpi radialis brevis muscle. We obtained the average of three measurements [30].
    The first point at which a pressure feeling turns into a pain sensation was determined using a pressure algometer (Wagner Instruments, Greenwich, USA, PainTestTM FPN 100 Algometer). Using a flat, circular, metal probe with a rubber covering, induced pressure was applied to the locations mentioned before. The algometer was mounted vertically and the pressure increased. Patients were asked to inform the examiner as soon as they felt any pain. Each measure was followed by a 30-second break. More sensitivity is indicated by lower PPT values [31].
  - Central Sensitization Inventory (CSI) [32]:
    The CSI was created in 2012 as a screening method to determine whether the symptoms being experienced are due to central sensitization or reflect central sensitivity [33]. Together with its initial construct validity, the CSI’s psychometric potency and clinical value are satisfactory. There are 25 items in the finished version. CSI values are calculated to a maximum of 100 points and range from 0 to 4. Subclinical (0–29), mild (30–39), moderate (40–49), severe (50–59), and extreme (60–100) are the five levels [34].
3. Physical Function Tests
  - Knee injury and Osteoarthritis Outcome Score (KOOS) [35]:
    The 42 items that make up the KOOS’s five subscales are pain, other symptoms, activities of daily living (ADL), sport and recreation, and knee-related quality of life. Each question receives a score between 0 and 4, which is converted to a score between 0 and 100. More difficulties are indicated by a lower score [35].
  - Timed up and go:
    This test measures how long it takes someone to get up from a regular chair, go three meters away to a line, turn around, and then get back in the chair and sit down. If necessary, the chair’s arms can be used as support while standing or sitting. On average, two trials were required [36]. This test and functional mobility have a good association. Healthy older people usually finish the task within just 10 s [37, 38].
  - Performance-based Chair Stand Test (CST):
    CST was carried out following the instructions in the Osteoarthritis Initiative manual. The patients were comfortably seated on the floor with their knees flexed a little beyond 90 degrees in a chair without armrests. Patients were instructed to rise five times as soon as they could without using their hands while using a stopwatch. After a countdown from three, time began at “Go” and finished at the fifth stand [39]. The reference value for the CST was 8.50 s (95% CI = 7.93–9.07 s) [40].
4. PainDETECT questionnaire (PD-Q) [41]:
  This is a neuropathic pain screening test. Its specificity and sensitivity were first claimed to be 80% and 85%, respectively, for the detection of neuropathic pain caused by back pain [41] and OA [30, 42]. Seven sensory-weighted descriptive questions and two questions describing the temporal and propagation aspects of pain make up this test. Scores between 13 and 18 imply undetermined points; the ultimate score ranges from 0 to 38 likelihood. For neuropathic pain, a score of ≤ 12 indicates low probability, whereas a score of ≥ 19 indicates high probability [41].
5. DN4 questionnaire [43]:
  The Douleur Neuropathique 4 questionnaire was created to evaluate neuropathic pain. There are ten questions, and the answers are yes or no. Seven of these items evaluate the intensity of the pain, while the remaining three, depending on the clinical examination, identify the presence of sensory allodynia and touch-needle hypoesthesia [43]. Each item answered as “yes” yields 1 point, and a total score at or above 4/10 is considered positive. This questionnaire has 83% sensitivity and 90% specificity for chronic pain associated with a lesion in the nervous system (central or peripheral) [44].
6. Hospital anxiety depression scale (HADS) [45]:
  The HADS was created to evaluate a patient’s level of anxiety and depression as well as the intensity change over time. It includes subscales for depression and anxiety. It consists of 14 different parts in total. Seven of these items (with odd numbers) evaluate anxiety, and the remaining eight (with even numbers) evaluate depression. An overall subscale score of > 8 points out of 21 indicates significant anxiety or depressive symptoms [45].
7. Radiological imaging:
  The Kellgren-Lawrence (KL) classification was originally described using AP knee radiographs. Each radiograph was given a score between 0 and 4 that corresponded to the degree of OA, with grade 0 denoting the absence of OA and grade 4 denoting severe OA [46].

### Statistical analysis

Microsoft Excel software was used to enter data collected throughout history, basic clinical examination, and outcome measures. Data was then imported into the Statistical Package for the Social Sciences (SPSS) version 22.0 software system for analysis. We tested the normality of the data using the Shapiro-Wilk test. Continuous variables were represented by mean and standard deviation (SD), and categorical variables were presented as frequencies and percentages (%). We tested the significance of associations between categorical variables using chi-square or Fisher’s exact tests (if > 20% of expected values were less than 5). A Mann‒Whitney test was used for continuous data, and a chi-square or Fisher’s exact tests were used for categorical data, to test for statistical significance between groups. This is because our data didn’t follow the normal distribution. Friedman’s test compares the mean ranks between the related groups and indicates how the groups differ. Significance between periods was measured by Dunn’s post hoc test. A multiple logistic regression analysis was conducted to assess the association between dependent and independent variables. Significant results were defined when the *p*-value was below 0.05.

We calculated the sample size where the effect size d was 0.71, with 80% power, a 95% confidence interval, and an error margin of 5%. The minimum required sample size was calculated to be 34 patients per group using G*Power 3.1.9.7 [47]. Participants were randomly assigned into two groups with 34 patients each: group 1 (active tVNS) and group 2 (sham tVNS).

## Results

We screened a total of 106 patients for inclusion and exclusion criteria. Of these potential participants, 38 were excluded as they did not meet the inclusion criteria. The reasons for exclusion were the presence of autoimmune diseases (*n* = 20) and refusal to enter the study (*n* = 18), leaving 68 patients to complete the study. Those 68 patients were randomly allocated to an active tVNS group (34 patients) and a sham tVNS group (34 patients).

Table 1 presents the sociodemographic characteristics of the study participants. A total of 68 patients, 4 males (5.8%), and 64 females (94.2%) were included in the study. The age of the patients ranged from 29 to 71 (mean 48.38 ± 10.0) for the active t-VNS group and from 32 to 70 (mean 45.85 ± 10.9) for the sham group. Both groups were comparable in age, sex, marital status, educational level, occupation, residence, disease duration, and body mass index (BMI), with a *p*-value > 0.05.

**Table 1:** Sociodemographic and personal characteristics of the study participants (*n*=68)

Variables	Active t-VNS group (*n*=34) N (%)	Sham t-VNS group (*n*=34) N (%)	*P* value*
**Age (Years)**			
**Mean ±SD**	48.38± 10.0	45.85± 10.9	0.325
**Range**	29-71	32-70	
**Sex**			
**Male**	2(5.8)	2(5.8)	0.693
**Female**	32(94.2)	32(94.2)	
**Symptoms Duration (years)**			
**Mean ±SD**	5.9±4.7	4.6±4.9	0.279
**Range**	0.16 - 20	1 - 17	
**Body mass index (kg/m**^**2**^**)**			
**Mean ±SD**	29.6±4.45	30.7±4.9	0.350
**Range**	23.0 - 44.5	25 - 40	
**Education**			
** No formal education**	6(17.6)	12(35.3)	0.199
** Middle (Secondary school or less)**	16(47.1)	10(29.4)	
**High (University or above)**	12(35.3)	12(35.3)	
**Occupation**			
** Housewives**	15(44.1)	24(70.6)	0.098
** Employees**	10(29.4)	4(11.8)	
** Manual workers**	1(2.9)	0(0.0)	
** Professionals**	8(23.5)	6(17.6)	
**Marital status**			
** Married**	26(76.5)	29(85.3)	0.731
** Widowed**	4(11.8)	2(5.9)	
** Divorced**	3(8.8)	3(8.8)	
** Single**	1(2.9)	0(0.0)	
**Residence**			
** Urban**	19(55.9)	13(38.2)	0.224
** Rural**	15(44.1)	21(61.8)	
**Chronic diseases**			
** No chronic diseases**	18(52.9)	25(73.5)	0.204
** HTN**	10(29.5)	6(17.6)	
** HCV**	6(17.7)	3(8.8)	

*HTN* Hypertension, *HCV* Hepatitis C virus

*Statistically significant at *p*-value < 0.05. Data presented as frequency (%) or mean ± SD and range

The overall study subjects’ VAS ranged from 5 to 10 (mean 8.29 ± 1.55), and their PD-Q mean was 15.4 ± 7.9. The means for DN4 and CSI were 3.28 ± 2.17 and 53.16 ± 13.93, respectively. With a range of 11 to 70, the mean of the pain subscale of the KOOS score was 29.1 ± 11.12.

The mean VAS score was 8.32 ± 1.45 for the active group and 8.26 ± 1.65 for the sham group. Mean scores for CSI were 55.76 ± 15.67 in the active group and 50.56 ± 11.59 in the sham group. As observed in Table 2, there were no statistically significant differences between the two groups for any of the baseline measurements, except for the KL scale (*P* = 0.044).

**Table 2:** Baseline pain and central sensitization measurements of both groups (*n*=68)

Variables pre-intervention	Active group (*n*=34) Mean±SD	Sham group (*n*=34) Mean±SD	*P* value
**VAS**	8.32±1.451	8.26±1.657	0.890
**Range**	5-10	5-10	
**PPT Rt knee (N)**	21.94±4.499	20.76±7.152	0.099
**PPT Lt knee (N)**	20.56± 5.615	19.41±9.860	0.494
**PPT Rt elbow (N)**	20.56±5.615	19.41±9.860	0.691
**CSI**	55.76±15.679	50.56±11.595	0.129
**KOOS total**	30.15±10.474	28.56±9.510	0.721
**KOOS symptoms**	39.56±16.382	38.79±14.384	0.916
**KOOS pain**	29.56±13.189	28.65±8.762	0.702
**KOOS ADL**	35.03±17.018	32.00±10.171	0.835
**KOOS sports**	10.88±12.938	8.09±13.372	0.291
**KOOS QOL**	35.71±13.386	34.56±19.109	0.578
**Timed up and go**	23.29±5.469	23.03±5.546	0.995
**CST**	24.91±6.571	22.71±3.842	0.063
**PainDETECT**	24.91±6.571	22.71±3.842	0.213
**DN4**	3.65±2.398	2.91±1.881	0.357
**HAD-D**	15.32±5.341	15.03±5.294	0.795
**HAD-A**	17.53±5.473	16.09±4.641	0.233
**KL score **			
1	0(0)	6(17.6)	
2	15(44.1)	16(47.1)	0.044
3	12(35.3)	6(17.6)	
4	7(20.6)	6(17.6)	

*VAS* Visual Analog Scale, *PPT* Pressure pain threshold, *N* newton, *CST* performance-based chair and stand test, *DN4* Douleur neuropathique 4, *HAD-A* Hospital Anxiety score, *HAD-D* Hospital Depression score, *CSI* Central sensitization inventory, *KL* Kellgren-Lawrence grading, *KOOS* Knee Injury and Osteoarthritis Outcome Score, *ADL* Activity of daily living, *QOL* Quality of life

### VAS

Compared to baseline levels, the immediate post-intervention VAS score for knee pain was significantly reduced in both the active and sham groups (*P* < 0.001). In addition, this reduction was maintained after 4 weeks post-intervention from baseline levels in both groups (*P* < 0.001) as demonstrated in Tables 3 and 4. In terms of the change in VAS score (Table 5), the improvement was significantly higher in the active group compared to sham tVNS immediately post-intervention (*P* < 0.001) and at 4 weeks post-intervention (*P* < 0.001).

**Table 3:** Comparison between the three studied periods according to different measurements in the active tVNS group (*n* = 34)

	Before intervention	Immediately post-intervention	4 weeks post-intervention	Fr (*p*)	Sig. bet. periods.		
					*p*_1_	*p*_2_	*p*_3_
**VAS**							
Mean ± SD.	8.32 ± 1.45	4.12 ± 1.68	4.24 ± 1.74	Fr = 64.235^*^ (< 0.001^*^)	< 0.001^*^	< 0.001^*^	0.716
Median (IQR)	8.0 (8.0–10.0)	4.0 (4.0–5.0)	4.0 (4.0–5.0)				
**PPT right knee**							
Mean ± SD.	21.94 ± 4.50	25.09 ± 8.66	24.91 ± 8.56	Fr = 10.750^*^ (0.005^*^)	0.011^*^	0.029^*^	0.716
Median (IQR)	22.0 (19.0–25.0)	30.0 (18.0–30.0)	30.0 (18.0–30.0)				
**PPT left knee**							
Mean ± SD.	20.56 ± 5.61	25.18 ± 4.62	25.21 ± 4.37	Fr = 11.840^*^ (0.003^*^)	0.015^*^	0.008^*^	0.808
Median (IQR)	20.0 (18.0–22.0)	25.0 (21.0–30.0)	25.0 (21.0–30.0)				
**PPT right Elbow**							
Mean ± SD.	21.0 ± 3.73	23.15 ± 6.27	23.32 ± 6.25	Fr = 13.872^*^ (0.001^*^)	0.039^*^	0.002^*^	0.332
Median (IQR)	21.0 (19.0–24.0)	24.0 (20.0–26.0)	25.0 (20.0–26.0)				
**CSI**							
Mean ± SD.	55.76 ± 15.68	26.18 ± 14.04	27.15 ± 13.68	Fr = 57.736^*^ (< 0.001^*^)	< 0.001^*^	< 0.001^*^	0.544
Median (IQR)	56.0 (46.0–72.0)	22.0 (19.0–40.0)	22.0 (20.0–40.0)				
**KOOS**							
Mean ± SD.	30.15 ± 10.47	61.59 ± 14.70	61.12 ± 14.21	Fr = 57.902^*^ (< 0.001^*^)	< 0.001^*^	< 0.001^*^	0.332
Median (IQR)	26.50 (25.0–36.0)	66.0 (45.0–75.0)	67.0 (45.0–73.0)				
**Timed up and go**							
Mean ± SD.	23.29 ± 5.47	13.32 ± 3.95	13.97 ± 3.90	Fr = 61.492^*^ (< 0.001^*^)	< 0.001^*^	< 0.001^*^	0.021^*^
Median (IQR)	25.0 (18.0–27.0)	12.0 (10.0–13.0)	13.0 (10.0–15.0)				
**CST**							
Mean ± SD.	24.91 ± 6.57	16.21 ± 3.08	16.59 ± 3.04	Fr = 60.372^*^ (< 0.001^*^)	< 0.001^*^	< 0.001^*^	0.146
Median (IQR)	24.0 (20.0–27.0)	16.0 (14.0–20.0)	16.50 (15.0–20.0)				
**PD-Q**							
Mean ± SD.	16.44 ± 7.84	7.82 ± 5.45	8.53 ± 5.50	Fr = 56.495^*^ (< 0.001^*^)	< 0.001^*^	< 0.001^*^	0.090
Median (IQR)	18.50 (10.0–21.0)	8.0 (3.0–13.0)	8.0 (3.0–13.0)				
**DN4**							
Mean ± SD.	3.65 ± 2.40	1.50 ± 1.40	1.56 ± 1.40	Fr = 48.805^*^ (< 0.001^*^)	< 0.001^*^	< 0.001^*^	0.808
Median (IQR)	4.0 (2.0–5.0)	1.0 (0.0–2.0)	1.0 (0.0–2.0)				
**HAD-D**							
Mean ± SD.	15.32 ± 5.34	7.44 ± 4.63	7.76 ± 4.42	Fr = 58.336^*^ (< 0.001^*^)	< 0.001^*^	< 0.001^*^	0.396
Median (IQR)	17.0 (11.0–20.0)	7.0 (4.0–11.0)	7.50 (4.0–11.0)				
**HAD-A**							
Mean ± SD.	17.53 ± 5.47	8.59 ± 4.43	8.88 ± 4.26	Fr = 58.074^*^ (< 0.001^*^)	< 0.001^*^	< 0.001^*^	0.332
Median (IQR)	18.0 (15.0–21.0)	8.50 (5.0–11.0)	9.0 (5.0–11.0)				
**CSI**							
Mean ± SD.	55.76 ± 15.68	26.18 ± 14.04	27.15 ± 13.68	Fr = 57.736^*^ (< 0.001^*^)	< 0.001^*^	< 0.001^*^	0.544
Median (IQR)	56.0 (46.0–72.0)	22.0 (19.0–40.0)	22.0 (20.0–40.0)				
**KOOS**							
Mean ± SD.	30.15 ± 10.47	61.59 ± 14.70	61.12 ± 14.21	Fr = 57.902^*^ (< 0.001^*^)	< 0.001^*^	< 0.001^*^	0.332
Median (IQR)	26.50 (25.0–36.0)	66.0 (45.0–75.0)	67.0 (45.0–73.0)				

*IQR* Inter quartile range, *SD* Standard deviation

Fr: Friedman test, Sig. bet. periods were done using Post Hoc Test (Dunn’s)

p: *p* value for comparing between the studied periods

p_1_: *p* value for comparing between Before intervention and Immediately post intervention

p_2_: *p* value for comparing between Before intervention and 1-month post intervention

p_3_: *p* value for comparing between Immediately post intervention and 1-month post intervention

*VAS* Visual Analog Scale, *PPT* Pressure pain threshold, *CST* Performance-based chair and stand test, *PD-Q* PainDETECT Questionnaire, *DN4* Douleur neuropathique 4, *HAD-A* Hospital Anxiety score, *HAD-D* Hospital Depression score, *CSI* Central sensitization inventory, *KL* Kellgren-Lawrence grading, *KOOS* Knee Injury and Osteoarthritis Outcome Score

*Statistically significant at *p* ≤ 0.05

### Central sensitization measures

#### PPT

The immediate post-intervention PPT-Rt knee was significantly increased compared to baseline in the active tVNS group (*P* = 0.011) and in the sham group (*P* < 0.001). This increase was maintained until 4 weeks post-intervention from baseline levels only in the active group (*P* = 0.029). Following the intervention (*P* = 0.016) and four weeks later (*P* = 0.003), the active group’s change was substantially higher than the sham group’s change (Table 5).

Regarding PPT-Lt. knee, the post-intervention measurement was significantly higher than the baseline in active tVNS. This improvement in active tVNS was maintained until 4 weeks post-intervention compared with baseline levels (*P* = 0.008). In contrast, in the sham group, there was no significant improvement immediately or after 4 weeks (Table 4). Four weeks after the intervention, the active group’s change was greater than the sham group’s (*P* = 0.002).

**Table 4:** Comparison between the three studied periods according to different measurements in the sham tVNS group (*n* = 34)

	Before intervention	Immediately post intervention	4 weeks post intervention	Fr (*p*)	Sig. bet. periods.		
					*p*_1_	*p*_2_	*p*_3_
**VAS**							
Mean ± SD.	8.26 ± 1.66	21.65 ± 7.11	20.76 ± 7.15	Fr = 46.621^*^ (< 0.001^*^)	< 0.001^*^	< 0.001^*^	1.000
Median (IQR)	9.0 (8.0–10.0)	20.0 (20.0–25.0)	20.0 (19.0–22.0)				
**PPT right knee**							
Mean ± SD.	20.76 ± 7.15	21.65 ± 7.11	20.76 ± 7.15	Fr = 36.0^*^ (< 0.001^*^)	0.001^*^	1.000	0.001^*^
Median (IQR)	20.0 (19.0–22.0)	20.0 (20.0–25.0)	20.0 (19.0–22.0)				
**PPT left knee**							
Mean ± SD.	19.41 ± 9.86	19.59 ± 9.93	19.41 ± 9.86	Fr = 4.000 (0.135)	–	–	–
Median (IQR)	20.0 (11.0–21.0)	20.0 (10.0–22.0)	20.0 (11.0–21.0)				
**PPT right Elbow**							
Mean ± SD.	20.94 ± 7.17	20.06 ± 7.56	20.94 ± 7.17	Fr = 4.000 (0.135)	–	–	–
Median (IQR)	22.0 (19.0–25.0)	20.0 (20.0–25.0)	22.0 (19.0–25.0)				
**CSI**							
Mean ± SD.	50.56 ± 11.59	47.12 ± 11.07	48.18 ± 12.13	Fr = 42.0^*^ (< 0.001^*^)	< 0.001^*^	< 0.001^*^	0.363
Median (IQR)	55.0 (46.0–58.0)	50.0 (40.0–57.0)	50.0 (40.0–57.0)				
**KOOS**							
Mean ± SD.	28.56 ± 9.51	28.56 ± 9.51	28.56 ± 9.51	Fr = 0.000 (1.000)	–	–	–
Median (IQR)	30.0 (24.0–32.0)	30.0 (24.0–32.0)	30.0 (24.0–32.0)				
**Timed up and go**							
Mean ± SD.	23.03 ± 5.55	22.97 ± 6.06	23.38 ± 5.88	Fr = 5.360 (0.069)	–	–	–
Median (IQR)	22.0 (20.0–28.0)	20.0 (20.0–30.0)	23.0 (20.0–30.0)				
**CST**							
Mean ± SD.	22.71 ± 3.84	22.09 ± 4.12	22.44 ± 4.05	Fr = 7.200^*^ (0.027^*^)	0.029^*^	0.275	0.275
Median (IQR)	22.0 (21.0–24.0)	20.0 (20.0–24.0)	22.0 (20.0–25.0)				
**PD-Q**							
Mean ± SD.	14.35 ± 7.95	13.47 ± 7.28	13.74 ± 7.29	Fr = 22.800^*^ (< 0.001^*^)	0.006^*^	0.102	0.275
Median (IQR)	15.0 (10.0–22.0)	15.0 (9.0–20.0)	15.0 (10.0–20.0)				
**DN4**							
Mean ± SD.	2.91 ± 1.88	2.91 ± 1.88	2.91 ± 1.88	Fr = 0.000 (1.000)	–	–	–
Median (IQR)	3.0 (1.0–5.0)	3.0 (1.0–5.0)	3.0 (1.0–5.0)				
**HAD-D**							
Mean ± SD.	15.03 ± 5.29	14.09 ± 5.18	14.88 ± 5.39	Fr = 15.071^*^ (0.001^*^)	0.011^*^	0.856	0.006^*^
Median (IQR)	15.0 (11.0–20.0)	15.0 (10.0–20.0)	16.0 (10.0–20.0)				
**HAD-A**							
Mean ± SD.	16.09 ± 4.64	14.09 ± 5.18	14.88 ± 5.39	Fr = 16.889^*^ (< 0.001^*^)	< 0.001^*^	0.146	0.029^*^
Median (IQR)	17.0 (15.0–20.0)	15.0 (10.0–20.0)	16.0 (10.0–20.0)				

*IQR* Inter quartile range, *SD* Standard deviation

Fr: Friedman test, Sig. bet. periods were done using Post Hoc Test (Dunn’s)

p: *p* value for comparing between the studied periods

p_1_: *p* value for comparing between Before intervention and Immediately post intervention

p_2_: *p* value for comparing between Before intervention and 1-month post intervention

p_3_: *p* value for comparing between Immediately post intervention and 1-month post intervention

*VAS* Visual Analog Scale, *PPT* Pressure pain threshold, *CST* Performance-based chair and stand test, *PD-Q* PainDETECT Questionnaire, *DN4* Douleur neuropathique 4, *HAD-A* Hospital Anxiety score, *HAD-D* Hospital Depression score, *CSI* Central sensitization inventory, *KL* Kellgren-Lawrence grading, *KOOS* Knee Injury and Osteoarthritis Outcome Score

*Statistically significant at *p* ≤ 0.05

**Table 5:** Comparison between Active tVNS and Sham tVNS according to change from baseline in different measurements (*n* = 68)

**Immediate post-intervention**				**4-weeks post-intervention**		
	**Active tVNS(***n*** = 34)**	**Sham tVNS(***n*** = 34)**	***p***	**Active tVNS(***n*** = 34)**	**Sham tVNS(***n*** = 34)**	***p***
**Decrease in VAS**						
Mean ± SD.	4.21 ± 1.92	1.50 ± 1.19	<0.001^*^	4.09 ± 1.88	1.53 ± 1.31	<0.001^*^
Median (IQR)	4 (3 – 5)	1 (1 – 2)		4 (3 – 5)	1 (0 – 3)	
**Increase in PPT right knee**						
Mean ± SD.	3.15 ± 8.26	0.88 ± 1.01	0.016^*^	2.97 ± 8.12	0.0 ± 0.0	0.003^*^
Median (IQR)	6 (-1 – 8)	1 (0 – 2)		6 (-1 – 8)	0 (0 – 0)	
**Increase in PPT left knee**						
Mean ± SD.	4.62 ± 7.55	0.18 ± 1.64	0.004^*^	4.62 ± 7.55	0.18 ± 1.64	0.002^*^
Median (IQR)	5 (-1 – 10)	0 (-1 – 0)		5 (-1 – 10)	0 (-1 – 0)	
**Increase in PPT right elbow**						
Mean ± SD.	2.15 ± 6.16	-0.88 ± 2.03	0.001^*^	2.32 ± 6.02	0.0 ± 0.0	<0.001^*^
Median (IQR)	2 (0 – 6)	0 (-2 – 0)		3 (0 – 6)	0 (0 – 0)	
**Decrease in CSI**						
Mean ± SD.	29.59 ± 14.14	3.44 ± 3.14	<0.001^*^	28.62 ± 13.55	2.38 ± 2.93	<0.001^*^
Median (IQR)	31(15 –37)	2 (0 – 7)		30 (15–38)	1 (0 – 6)	
**Increase in KOOS**						
Mean ± SD.	31.44 ± 18.49	0.0 ± 1.28	<0.001^*^	30.97 ± 18.22	0.0 ± 0.0	<0.001^*^
Median (IQR)	39(14 – 47)	0 (-1 – 1)		38(14 – 45)	0 (0 – 0)	
**Decrease in timed up and go test**						
Mean ± SD.	9.97 ± 6.05	0.06 ± 1.25	<0.001^*^	9.32 ± 5.87	-0.35 ± 0.88	<0.001^*^
Median (IQR)	11 (4 – 14)	0 (-1 – 0)		9.50 (3 – 13)	0 (-1 – 0)	
**Decrease in CST**						
Mean ± SD.	8.71 ± 6.45	0.62 ± 1.07	<0.001^*^	8.32 ± 6.28	0.26 ± 0.96	<0.001^*^
Median (IQR)	7 (5 – 10)	1 (0 – 2)		7 (4 – 10)	0 (-1 – 1)	
**Decrease in PD-Q**						
Mean ± SD.	8.62 ± 7.19	0.88 ± 1.09	<0.001^*^	7.91 ± 6.61	0.62 ± 1.07	<0.001^*^
Median (IQR)	7 (4 – 11)	0 (0 – 2)		6 (4 – 9)	0 (0 – 2)	
**Decrease in DN4**						
Mean ± SD.	2.15 ± 2.02	0.01± 0.0	<0.001^*^	2.09 ± 1.90	0.0 ± 0.08	<0.001^*^
Median (IQR)	2 (0 – 3)	0 (0 – 0)		2 (0 – 3)	0 (0 – 0)	
**Decrease in HAD-D**						
Mean ± SD.	7.88 ± 4.31	0.94 ± 1.63	<0.001^*^	7.56 ± 3.86	0.15 ± 1.05	<0.001^*^
Median (IQR)	7 (5 – 10)	1 (0 – 1)		7 (5 – 10)	0 (0 – 0)	
**Decrease in HAD-A**						
Mean ± SD.	8.94 ± 5.72	2.0 ± 2.63	<0.001^*^	8.65 ± 5.30	1.21 ± 2.52	<0.001^*^
Median (IQR)	8 (6 – 10)	2 (0 – 4)		8 (6 – 10)	1 (0 – 2)	

*SD* Standard deviation

p: *p* value for comparing between Active tVNS and Sham tVNS using Mann Whitney test

*VAS* Visual Analog Scale, *PPT* pressure pain threshold, *CST* performance-based chair and stand test, *PD-Q* PainDETECT Questionnaire, *DN4* Douleur neuropathique 4, *HAD-A* Hospital Anxiety score, *HAD-D* Hospital Depression score, *CSI* Central sensitization inventory, *KL* Kellgren-Lawrence grading, *KOOS* Knee Injury and Osteoarthritis Outcome Score

*Statistically significant at *p* ≤ 0.05

Comparing the immediate post-intervention PPT-Rt elbow to baseline, there was a significant increase in active tVNS (*P* = 0.039). This improvement in active tVNS continued until 4 weeks post-intervention compared to baseline levels (*P* = 0.002). In contrast, there was no statistically significant improvement in the sham group either right after the intervention or until four weeks later (*P* = 0.135) (Tables 3 and 4).

#### CSI

Regarding the CSI, its mean improved significantly in the active tVNS group immediately post-intervention (*P* < 0.001) but increased insignificantly to 27.15 ± 13.68 at the end of the follow-up period. However, this improvement was maintained until the end of the follow-up. Additionally, there was a significant decrease in the sham group from 50.56 ± 11.59 to 47.12 ± 11.07, *P* < 0.001 immediately post-intervention but not maintained at the second visit. As displayed in Table 5, the active group’s improvement was substantially greater than the sham group’s change both immediately and four weeks after the intervention (*P* < 0.001).

### Physical function measurements

Regarding the difference in KOOS score, the mean increased significantly from 30.15 ± 10.47 in active tVNS at baseline to 61.59 ± 14.70 immediately post-intervention (*P* < 0.001). Besides, this improvement was maintained until the end of the follow-up (*P* < 0.001). In contrast, there was no significant change in the sham group from baseline to the immediate post-intervention visit and at the second visit levels (*P* = 1.000).

The timed up and go test showed a significant drop from 23.29 ± 5.47 in active tVNS at baseline to 13.32 ± 3.95 immediately post-intervention (*P* < 0.001). Additionally, the improvement was maintained until the end of the study (*P* = 0.021). On the other hand, there was no significant change in the sham group (*P* = 0.069). On top of that, the active group’s mean score on the chair and stand test dropped from 24.91 ± 6.57 at baseline to 16.21 ± 3.08 upon completion of the intervention (*P* < 0.001), and this improvement persisted until the end of the follow-up (*P* < 0.001), while did not (*P* = 0.275) in the sham group, as demonstrated in Tables 3 and 4. Furthermore, both immediately after the intervention and four weeks later, the active group’s improvement was considerably greater than the sham group’s (*P* < 0.001) as illustrated in Table 5.

### Neuropathic pain measures

#### PD-Q

Compared to baseline levels, the immediate post-intervention PD-Q score was significantly reduced in the active tVNS group (*P* > 0.001) and the sham group (*P* = 0.006). In addition, this reduction in score was maintained until 4 weeks post-intervention in active tVNS (8.53 ± 5.50, *P* < 0.001) but not in sham tVNS (13.74 ± 7.29, *P* = 0.102). The improvement of the active group was significantly greater than that of the sham group after the intervention and four weeks later (*P* < 0.001) (Table 5).

#### DN4

The immediate post-intervention DN4 score was significantly reduced compared to baseline levels in active tVNS (*P* < 0.001). Moreover, this reduction was maintained until 4 weeks post-intervention in the active group (1.56 ± 1.40, *P* < 0.001). However, this was not the case in sham tVNS, where there was no significant difference during the study period (*P* = 1.000).

### Hospital anxiety depression scale

Regarding the difference in depression scores, the mean decreased significantly in the active tVNS group immediately post-intervention (*P* < 0.001). Furthermore, this improvement was maintained till the end of the follow-up (*P* < 0.001). Although there was a significant decrease in the sham group (*P* = 0.011) immediately following the intervention, this change wasn’t maintained at the second visit (*P* = 0.856) as shown in Tables 3 and 4.

When comparing the improvement in the depression scale, the immediate post-intervention improvement was considerably higher in the active tVNS group than in the sham group (7.88 ± 4.31 vs. 0.94 ± 1.63, respectively, *P* < 0.001). Furthermore, after 4 weeks post-intervention, active tVNS showed a significantly greater decrease from baseline levels than sham tVNS (7.56 ± 3.86 vs. 0.15 ± 1.05, respectively, *P* < 0.001), as illustrated in Table 5.

Meanwhile, as shown in Table 6, there was a significant difference between both groups regarding the number of cases that achieved the minimum clinically important difference (MCID) (*P* > 0.001). It is considered that MCID In KOOS is 10 [48], 1.8 for the HADS Anxiety subscale, and 1.5 for the Depression subscale [49]. Further, we estimated that the number needed to treat (NNT) for the previously mentioned scores was approximately 2.

Regarding adverse effects, 60 participants (88%) experienced skin irritation, while 55 participants (80.8%) reported tingling at the location of the tVNS application. These side effects were mild and tolerable, and no drop-off occurred due to those side effects.

Finally, a multivariate linear regression was run to predict the 4-week percent improvement (from baseline) in VAS in the active group. The PPT over the right knee was the only significant predictor, with *P* = 0.002. Additionally, PPT over the right knee in the univariate model and CST in the multivariate model were the only significant predictors of the 4-week percent improvement (from baseline) in the KOOS score in the active group, *P* = 0.042 and *P* = 0.013, respectively.

**Table 6:** Comparison between active tVNS and sham tVNS according to the number of cases achieved the MCID in different measurements with NNT

	Active tVNS (*n* = 34)	Sham tVNS (*n* = 34)	χ^2^	*p*	NNT
KOOS [Change > 10]	26 (76.5%)	0 (0.0%)	42.095^*^	< 0.001^*^	1.3 ≈ 2
HAD-A [Change > 1.8]	32 (94.1%)	9 (26.5%)	32.495^*^	< 0.001^*^	1.5 ≈ 2
HAD-D [Change > 1.5]	32 (94.1%)	3 (8.8%)	49.513^*^	< 0.001^*^	1.2 ≈ 2
Timed up and go test [Change > 3.4]	25 (73.5%)	0 (0.0%)	39.535^*^	< 0.001^*^	1.4 ≈ 2

χ^2^: Chi square test

p: *p* value for comparing between Active tVNS and Sham tVNS

*HAD-A* Hospital Anxiety score, *HAD-D* Hospital Depression score, *KOOS* Knee Injury and Osteoarthritis Outcome Score, *NNT* number needed to treat

*Statistically significant at *p* ≤ 0.05

## Discussion

This proof-of-concept feasibility trial aimed to assess the efficacy and safety of a 12-week tVNS treatment for knee OA. Considering tVNS’s efficacy on pain, inflammation, and central sensitization as well as its safety profile, we hypothesized that tVNS could be an effective line of management for knee OA. In addition, tVNS could be implemented easily in clinical practice and included in therapeutic sessions as trained medical personnel with appropriate settings are the only requirements.

We demonstrated four major findings: first, active tVNS improved all the tested parameters, and this improvement continued for 4 weeks after the end of the 3-month treatment period. Second, although in sham tVNS, the VAS score decreased and was maintained until the end of the follow-up period, improvement in the PPT right knee, PD-Q, CST, depression, anxiety, and CSI was not maintained through the end of the follow-up period. Third, the improvement from baseline until 4 weeks after intervention in all these parameters was significantly higher in the active group than in the sham group where *p* < 0.001 in all variables except for PPT right knee. (*P* = 0.003) and PPT left knee (*P* = 0.002). Fourth, the PPT left knee, PPT right elbow, DN4 questionnaire, timed up and go test, and KOOS score showed no improvement at all in the sham group.

### Nociceptive pain

Nociceptive pain, which can be classified as visceral (internal organs) or somatic (muscles, joints), results from real harm to non-neural tissue. Because somatic tissues have a high concentration of nociceptors, persistent somatic pain is usually localized and a result of degenerative processes (e.g., arthritis) [50]. We noticed that there was a significant improvement in the VAS score in the active tVNS group between the baseline and first follow-up visits and between the baseline and second follow-up visits. The active group’s mean VAS improvement was noticeably higher than that of the sham group. This analgesic effect on OA study participants’ pain is consistent with previous studies of VNS in other painful disorders. This preliminary report is the first to examine the potential anti-nociceptive effects of long-term tVNS in patients with knee OA. The stimulation is considered safe. The most common side effects are tingling and skin irritation at the stimulation site as reported by other studies [51].

In line with our findings, tVNS was applied for the first time to erosive hand osteoarthritis by Courties et al. [52]. According to their findings, the baseline VAS for hand discomfort was 60 mm [50; 78.2]. In 16/18 patients, tVNS considerably decreased the VAS score, with a median reduction of 23.5 mm [7.7; 37.2] [52].

Since there hasn’t been much previous study on the effectiveness of VNS in OA, we will talk about data from several conditions that share persistent pain and central sensitization with knee OA. Venborg et al. discovered that t-VNS caused a 14% reduction in the VAS score just for the hips on day 5 compared to the baseline (5.1 ± 2.8 vs. 4.4 ± 2.8, *p* = 0.04) in polymyalgia rheumatica patients [53]. Addorisio et al. also revealed a decrease in the VAS global health of the patient (approximately 20 mm on a 0–100 mm scale) in RA patients [54].

In addition, systemic lupus patients receiving active tVNS achieved a significantly superior reduction in their pain compared with sham (*P* = 0.049) in a study by Aranow and colleagues [55]. Besides, VNS ameliorated patient-reported pain intensity and anxiety in patients with chronic pelvic pain, as found by Napadow et al. [56]. This antinociceptive effect was also observed when VNS was applied by Kirchner et al. [57] on epileptic patients, Laqua et al. [58], and Janner et al. [59] on healthy volunteers.

In contrast, Muthulingam et al. [60] found that in patients with chronic pancreatitis, there were no differences in VAS scores after tVNS treatment compared to sham treatment (*P* = 0.7). However, active tVNS and sham treatments improved the maximum pain scores compared to their respective baselines (*P* = 0.007 for active, *P* = 0.018 for sham) [60]. This may be a result of the short treatment period and different pain mechanisms associated with chronic pancreatitis.

### Central sensitization

Our results showed that PPT in the diseased sites (both knees) impacted by peripheral and central sensitization improved markedly in the active tVNS group compared to baseline. The remote site (elbow) also showed a considerable improvement in PPT, which indicates the VNS influence on central sensitization. The improvement persisted for 4 weeks following the intervention, and PPT revealed a statistically larger improvement in the active group than in the sham group. These results point to the effect of VNS on both peripheral and central sensitization.

Similarly, Busch et al. [61] showed an increase in mechanical and pressure pain thresholds and a reduction in mechanical pain sensitivity after 1 h of continuous tVNS. In addition, Lange et al. found that there was a significant decrease in pain sensitivity following tVNS and during subsequent visits in patients with fibromyalgia [62]. A possible explanation for musculoskeletal pain reduction with VNS is to tune down mechanisms that stimulate central sensitization.

On the other hand, Juel et al. showed that, compared to sham stimulation, tVNS induced no demonstrable differences in muscle pressure thresholds or bone pressure thresholds [63]. According to these findings, VNS may not consistently affect pain perception but rather depend on the pain modes studied or the recipient’s emotional state at the time of stimulation. Despite their paradoxical nature, these findings support the theory that VNS analgesic effects are strongly influenced by individual sensitivity and stimulation settings.

It has been hypothesized that vagal modulation of nociplastic pain is responsible for tVNS’ observed clinical effects. Factors contributing to chronic pain and central sensitization as inflammation, sympathetic tone, and the pain neuromatrix are also inhibited through vagal stimulation [64]. In support of these theories, we demonstrated that patients receiving active tVNS improved significantly more than patients receiving sham tVNS.

It has been demonstrated consistently by Botha et al. [65] that deep breathing, which raises parasympathetic tone, decreases acid-induced oesophageal hypersensitivity when compared to sham breathing (*P* = 0.0001) using a validated model of acid-triggered oesophageal pain. Similarly, Farmer et al. [66] showed that t-VNS delayed the onset of acid-induced oesophageal hypersensitivity (*P* = 0.004). Furthermore, when compared to sham, active t-VNS reversed preexisting acid-induced oesophageal hypersensitivity.

In addition, Kirchner et al. [57] showed that VNS decreased both the increasing pain associated with trains of five successive stimuli spaced 1.5 s apart as well as the discomfort associated with tonic pressure. Consequently, VNS primarily reduced pain in experimental methods where central processing magnified the pain intensity, indicating that tVNS could be employed to manage pain hypersensitivity.

In light of these findings, it is suggested that tVNS is useful in managing pain hypersensitivity since it decreases pain in experimental methods where central processing intensifies pain.

### Physical function

Our results showed a significant improvement in physical function 4 weeks post-intervention in the active group as assessed by the total KOOS score compared to baseline. Furthermore, this improvement was maintained until the end of the follow-up (*P* < 0.001), unlike the sham group, which did not experience any notable changes. In line with our results, Courties et al. [52], showed a significant improvement in the Functional Index for Hand Osteoarthritis score in 14 of the 18 patients. This was associated with a median decrease of 2 points after treatment with tVNS.

### Neuropathic pain

As regards neuropathic pain, PD-Q and DN4 scores statistically improved in active tVNS, and this improvement was sustained through follow-up. In contrast, the DN4 questionnaire showed no improvement at all, while the PD-Q demonstrated improvement but wasn’t sustained at the end of follow-up in the sham group.

Our findings are supported by studies using different assessment methods. For example, the results shown by Kirchner et al. [67] and Thán et al. [68] indicated a significant influence of VNS on peripheral neurogenic inflammation. Further, Lyubashina et al. [69] observed that continuous cervical VNS suppressed (48%) or excited (29.5%) electrically evoked firing rates in spinal trigeminal nuclei neurons, but did not induce responses in 22.5%. Over 80% of respondents experienced prolonged suppression after stimulation ended, rather than facilitation.

Furthermore, Weissman-Fogel et al. [70] used a rat model of vincristine (VCR)-induced pain following sub-diaphragmatic vagotomy to investigate this potential contribution (SDV). They discovered that VCR + SDV rats developed painful neuropathy more rapidly than VCR alone rats, as shown by the following findings: (1) rats injected with SDV + VCR developed central sensitization earlier in the first week, whereas rats injected with VCR alone developed it only at the second week, (2) spontaneous pain was more common, and (3) mechanical dynamic allodynia was higher [70]. Based on these previous studies, decreased vagal activity aggravates both the severity and duration of central sensitization and neuropathic pain.

### Depression and anxiety

It has recently been proposed that part of the anti-nociceptive effect of VNS may be mediated by the VNS’ positive effects on emotional states. It is now recognized that OA pain is frequently accompanied by co-morbid affective manifestations, such as anxiety and depression, which could additionally aggravate pain perception [71]. Studies suggest that both tVNS and slow breathing improve the affective state; therefore, tVNS may produce beneficial effects in individuals with chronic pain [72].

The effect of VNS on noradrenergic and serotonergic neurons has also been proposed to be part of its beneficial effect. In multiple studies, VNS has been shown to increase noradrenaline concentrations and improve noradrenaline and serotonergic neurotransmission in brain regions important in depression [73–75]. Given that VNS can improve emotional state, this will achieve pain relief. Thus, mood enhancement is one of the mechanisms through which VNS can decrease pain.

Consistent with the previous studies, our results showed a statistically significant improvement in HADS immediately post-intervention in both the active and sham groups, but the improvement in the active group was significantly larger than that in the sham group. Similarly, Li et al. [76] found that the HADS-A and HADS-D in the tVNS group were lower than those in the control group from the first month onward in stroke patients. In addition, Kamourieh et al. tried VNS in eight chronic paroxysmal hemicrania patients and found a reduction in both HADS-A and HADS-D scores [77].

Also, Bajbouj et al. [78] reported that Among chronic “treatment-refractory depression” patients receiving VNS, 53.1% met the response criteria of a 50% decrease in the Hamilton Rating Scale for Depression (HRSD). In addition, 38.9% fulfilled the remission criteria on HRSD scores. While Lui et al. [79] and Rong et al. [80], reported that after tVNS, the 24-item Hamilton Depression Rating Scale scores’ reduction in the real tVNS group was significantly higher than in the sham tVNS group.

Finally, we found that the PPT over the right knee was the only significant predictor of 4-week percent improvements in VAS (from baseline) in the active group; this supports central sensitization’s role in osteoarthritis pain perception. It also points to the mechanism by which VNS achieves pain relief in OA. So, it is suggested that VNS may be more useful in centrally sensitized patients.

### Limitations

Our work has some limitations. First, a double-blind trial might have been more robust than a single-blind design, as double blindness would generate fewer errors. However, the sham-control design still provided reliable and strong results. Moreover, the fear of dropping out made the period of follow-up shorter.

Second, we did not use the entire set of quantitative sensory testing; instead, we used the central sensitization inventory, a valuable and valid tool for screening central sensitization, and a pressure algometer to measure pressure pain threshold. PPT has excellent reliability and demonstrated less variability than other QST measurements when used in knee OA patients and healthy participants [81].

Third, there is a lack of a standard of care arm that could offer more information about the effectiveness of VNS. Fourth, most of our cases were women, which may restrict the generalizability of our findings. Fifth, the KL scale showed a baseline significant difference (*P* = 0.044) which could be a source of bias and should be considered while interpreting the results. We consider these results to be preliminary and need to be confirmed by more studies using a larger sample over a longer period.

## Conclusion

The potential of tVNS is not limited to the treatment of depression and epilepsy. Instead, technology is being investigated for various disorders including migraine, tinnitus, inflammatory conditions, and pain. Currently, the exact pathways depicting the VNS mechanism of action on different types of pain are not well understood. Our research and other future research will contribute to developing the appropriate stimulation parameters. Considering its enormous potential therapeutic gain and high safety profile, further applications of VNS are encouraging.

Our findings provide evidence that 3 sessions weekly for 12 weeks of tVNS over the cymba concha can produce nociceptive and neuropathic pain relief as well as physical function and central sensitization improvement in knee OA patients. This study provides preliminary evidence for the beneficial effects of tVNS in OA and raises the possibility of using neuromodulation as an add-on to existing treatments.

## Supplementary Information

Supplementary Material 1.

Supplementary Material 2.

## Data Availability

The data that support the findings of this study are not openly available due to reasons of sensitivity and are available from the corresponding author upon reasonable request.

## Abbreviations

ABVN: Auricular Branch of The Vagus Nerve
ADL: Activity of Daily Living
BMI: Body mass index
CSI: Central Sensitization Inventory
CST: Performance-Based Chair Stand Test
DN4: Douleur Neuropathique 4 Questionnaire
HADS: Hospital Anxiety Depression Scale
HAD-A: Hospital Anxiety score
HAD-D: Hospital Depression score
HRSD: Hamilton Rating Scale for Depression
IL-1β: Interleukin-1 Β
IQR: Interquartile Range
KL: Kellgren-Lawrence Grading System
KOOS: Knee Injury and Osteoarthritis Outcome Score
MCID: Minimum Clinically Important Difference
NNT: Number Needed to Treat
OA: Osteoarthritis
PPT: Pressure Pain Threshold
QOL: Quality of Life
QST: Quantitative Sensory Testing
RA: Rheumatoid Arthritis
SD: Standard Deviations
SDV: Sub-diaphragmatic vagotomy
TENS: Transcutaneous Electrical Nerve Stimulation
TNF: Tumor Necrosis Factor
tVNS: Transcutaneous Vagus Nerve Stimulation
VAS: Visual Analog Scale
VCR: Vincristine
VNS: Vagus Nerve Stimulation
