# Supplementary material for: Transcutaneous vagus nerve stimulation as a pain modulator in knee osteoarthritis: a randomized controlled clinical trial
Source: BMC Musculoskelet Disord. 2025 Jan 20;26:68. doi: 10.1186/s12891-025-08288-6 (PMC11744843; doi:10.1186/s12891-025-08288-6)
Supplement: Supplementary file 1 — Supplementary Material 1. [file 12891_2025_8288_MOESM1_ESM.docx]

Table 1: side effects during the stimulation period in all participants (n = 86)

| Side effect | Frequency | Percentage |
| --- | --- | --- |
| Tingling | 55 | 80.8% |
| Skin irritation | 60 | 88% |
