# Supplementary material for: Transcutaneous vagus nerve stimulation as a pain modulator in knee osteoarthritis: a randomized controlled clinical trial
Source: BMC Musculoskelet Disord. 2025 Jan 20;26:68. doi: 10.1186/s12891-025-08288-6 (PMC11744843; doi:10.1186/s12891-025-08288-6)
Supplement: Supplementary file 2 — Supplementary Material 2. [file 12891_2025_8288_MOESM2_ESM.docx]

**Transcutaneous Vagus Nerve Stimulation as a Pain Modulator in Knee Osteoarthritis: a randomized controlled clinical trial**

**Investigators:**

**Gehad Gamal Elsehrawy^1^*****, Maha Emad Ibrahim^2^, Nermeen Hassan A.moneim^3^,**

**Mohamed Ahmed Hefny^4^, Nashwa Kamel El Shaarawy^5^**

1 Department of Physical Medicine, Rheumatology and Rehabilitation, faculty of medicine, Suez Canal University, Ismailia, 41522, Egypt; [gehadgamal@med.suez.edu.eg](mailto:gehadgamal@med.suez.edu.eg)

2 Department of Physical Medicine, Rheumatology and Rehabilitation, faculty of medicine, Suez Canal University, Ismailia, 41522, Egypt; [maha.ibrahim@med.suez.edu.eg](mailto:maha.ibrahim@med.suez.edu.eg)

3 Department of Physical Medicine, Rheumatology and Rehabilitation, faculty of medicine, Suez Canal University, Ismailia, 41522, Egypt; [dr.nermeen_hassan@med.suez.edu.eg](mailto:dr.nermeen_hassan@med.suez.edu.eg)

4 Department of Physical Medicine, Rheumatology and Rehabilitation, faculty of medicine, Suez Canal University, Ismailia, 41522, Egypt; [m.hefny@med.suez.edu.eg](mailto:m.hefny@med.suez.edu.eg)

5 Department of Physical Medicine, Rheumatology and Rehabilitation, faculty of medicine, Suez Canal University, Ismailia, 41522, Egypt; [Nashwa_saleh@med.suez.edu.eg](mailto:Nashwa_saleh@med.suez.edu.eg)

*Corresponding author:

**Gehad Gamal Elsehrawy**

Department of Physical Medicine, Rheumatology and Rehabilitation, Suez Canal University,

41522, Kilo 4.5 round road, Ismailia, Egypt

ORCID Number: https://orcid.org/0000-0002-1447-0543

Email: [gehadgamal@med.suez.edu.eg](mailto:gehadgamal@med.suez.edu.eg) or [dr.ggrheu@gmail.com](mailto:dr.ggrheu@gmail.com);

Tel: +20 1008162192

**Abstract**

**Background**

Our understanding of osteoarthritis (OA) has evolved from a degenerative disease to one in which low-grade, chronic inflammation plays a central role. In addition, evidence suggests that OA is accompanied by both peripheral and central nervous system sensitization that can cause pain. It has been demonstrated that transcutaneous vagus nerve stimulation (tVNS) can relieve pain, inflammation, and central sensitization in other conditions including fibromyalgia, pelvic pain, and headaches. We aimed to assess the efficacy and safety of tVNS on nociceptive pain, central sensitization, and physical function in knee OA.

**Methods**

In this 12-week study, we stimulated the auricular branch of the vagus nerve with an auricular electrode connected to a transcutaneous electrical nerve stimulation device once a day for 3 days each week for 12 weeks. A total of 68 patients with chronic knee OA were randomly assigned to the active and sham groups (34 patients in each group). We used a variety of outcome measures, including the visual analog scale (VAS), pressure pain threshold (PPT), knee injury and osteoarthritis outcome score (KOOS), PainDETECT (PD-Q) and Douleur Neuropathique 4 (DN4) questionnaires. Outcome measures were recorded at baseline, At the end of the stimulation period, and then after 4 weeks.

**Rational and background**

Osteoarthritis (OA) is the most common joint disorder in elderly individuals, affecting nearly 10% of men and 18% of women. As chronic pain and loss of function are common symptoms of OA, the socioeconomic burden is substantial. This burden costs in developed countries between 1.0% and 2.5% of gross domestic product [1]. Currently, no cure or treatment can prevent the destruction of joints. The Global Burden of Disease ranks OA of the hip and knee 11^th^ in terms of its contribution to worldwide disability [2].

Over the past decade, we have seen a fundamental shift in our understanding of the mechanisms that cause pain in osteoarthritis, in which inflammation and central sensitization play a crucial role. We are no longer viewing OA as just a degenerative disease but rather as a multifactorial disorder in which chronic low-grade inflammation plays a central role [3]. Old age, prior joint injuries, and obesity all contribute to joint damage, which triggers an immune response leading to chronic, low-grade inflammation and OA development [4].

Inflammation in OA is mediated by damage-associated molecular patterns, Toll-like receptors, the complement system, macrophages, and mast cells. Cytokines, interleukin-1 (IL-1β), tumor necrosis factor (TNF), chemokines, growth factors, adipokines, prostaglandins, leukotrienes, nitric oxide, and neuropeptides are also included in the inflammatory process associated with OA [5]. In addition, central nervous system pain pathways, including those governing descending inhibitions, may be affected by persistent stimulation of dorsal root ganglia by inflammatory cytokines originating in the joint and leading to central sensitization in chronic pain diseases like OA. As a result, evidence points to both peripheral and central nervous system sensitization as sources of pain in osteoarthritis [6].

Interestingly, it was found that stimulation of the afferent vagus nerve may activate the hypothalamic-pituitary-adrenal axis, resulting in an anti-inflammatory effect [7]. A disruption of this anti-inflammatory vagal reflex has been found in various autoimmune and inflammatory disorders, including rheumatoid arthritis (RA), pancreatitis [8], inflammatory bowel disease, and other conditions in which inflammation plays a role in the pathogenesis [9].

Moreover, pharmacological activation of nicotinic acetylcholine receptors (a7nAChR) or electrical stimulation of the cholinergic anti-inflammatory pathway by VNS may lower cytokine production, delay joint destruction, and ameliorate clinical signs of arthritis [10]. This cholinergic anti-inflammatory reflex can also be externally stimulated by implanting an external device close to the vagus nerve that delivers electrical impulses [11]. Furthermore, recent research demonstrated that VNS significantly reduced TNF and IL-6 production and lowered the severity of rheumatoid arthritis, even in some individuals with therapy-resistant illness [12]. Therapy with nicotine reduced TNF production in the colon and improved colitis, but subdiaphragmatic vagotomy of the ventral and dorsal vagus nerves raised the colitis disease activity score and markedly elevated TNF, IL-6, and IL-1b production in colon tissue [13].

Additionally, VNS is generally well tolerated and has been used to treat medication resistant epilepsy in more than 100,000 individuals [12]. With few adverse effects during electrical stimulation of the nerve, such as hoarseness, dysphonia, and coughing, it is a safe procedure and well tolerated by patients [14]. VNS is currently approved as a treatment for epilepsy, depression, and tinnitus, while it is also being researched as a potential novel treatment for stroke, RA, Crohn's disease, and heart failure [15].

Recent human and animal research shows mounting evidence that VNS can have potent analgesic benefits in addition to its anti-inflammatory effects. Inhibiting spinal nociceptive reflexes through vagal afferents may be beneficial for treating a variety of chronic pain syndromes, such as fibromyalgia, pelvic pain, and headaches [16].

Considering tVNS’s efficacy on pain, inflammation, and central sensitization as well as its safety profile, we hypothesized that tVNS could be a new treatment for knee OA, a subtype of chronic painful and inflammatory arthritis. This first proof-of-concept trial aimed to assess the safety and efficacy of tVNS on nociceptive pain, neuropathic pain, central sensitization, and physical function in individuals with knee OA.

**Aim of the Study**

The aim of this study was to assess the effect of the transcutaneous vagal nerve stimulation (tVNS) on pain in knee osteoarthritis patients.

**Study Objectives**

To evaluate the efficacy of transcutaneous vagal nerve stimulation in improving physical function, anxiety, and depression in patients with knee osteoarthritis.

**Materials and Methods**

We performed a single-blinded, sham-controlled, randomized clinical trial to assess the use of transcutaneous VNS as a pain modulator in patients with knee OA. It was a single-center trial that was carried out at the Physical Medicine, Rheumatology, and Rehabilitation outpatient clinics at Suez Canal University Hospitals in Ismailia, Egypt, between December 2019 and March 2022.

The study protocol was approved by the Committee of Ethical Research, Faculty of Medicine, Suez Canal University, (date of approval: 25/3/2019, Number 3905#). This was conducted according to the Deceleration of Helsinki and its subsequent modifications (193), and all patients provided written informed consent. The study was registered on ClinicalTrials.gov (NCT05387135) on 24/05/2022.

We recruited patients attending the Rheumatology and Rehabilitation outpatient clinics at Suez Canal University Hospital and who were diagnosed with knee OA based on the American College of Rheumatology (ACR) criteria [17]. Individuals were excluded if they had total knee arthroplasty, arthrodesis, autoimmune disease, or psychiatric disorders. Also, patients who had cellulites or skin ulceration at area of therapy application, implanted electrical devices, or on selective serotonin and norepinephrine-reuptake inhibitor drugs and anti-convulsant drugs were excluded.

We calculated the sample size where the effect size d was 0.71, with 80% power and a 95% confidence interval, and an error margin of 5%. The minimum required sample size was calculated to be 34 patients per group using G*Power 3.1.9.7 [18]. Participants were randomly assigned (1:1) into two groups with 34 patients each: group 1 (active tVNS) and group 2 (sham tVNS) using the order of entrance into the study.

**Experimental design:**

Participants were randomly allocated into two groups: active and sham. The duration of the tVNS treatment in this study was three months, and the number of stimulation sessions was decided upon in accordance with other research that demonstrated the effectiveness of tVNS in treating various chronic pain conditions [19]. Patients were blinded to group allocation, whereas the treating physician, who set the tVNS according to the protocol, was aware of the stimulation condition.

**Active treatment:**

A tVNS device (TENS 7000TM) made by Roscoe Medical Inc. was used to stimulate the afferents of the auricular branch of the vagus nerve (ABVN). The TENS 7000TM device, which comprises of a stimulator unit and a bipolar stimulation electrode, is described as a nerve stimulator and low-risk medical device (Instruction manual for TENS 7000), figures 1, 2. After being cleaned with an alcohol swab, the electrode was applied directly to the skin in the left cymba concha as shown in figure 3.

Prior to the experimental methods, subjects were accustomed to the stimulus for five minutes. The intensity was then gradually increased until the ideal intensity was obtained (i.e., clear tingling sensation, but not painful). Following that, patients are free to sit up or lie on their side. Due to habituation, the intensity was readjusted throughout the 30 min of continuous stimulation, aiming at the initial perceptive experience [20]. The stimulation for both groups lasted for 30 minutes once a day for 3 days per week for 12 weeks. The amplitude of the output current was between 0.25 and 2.0 mA as tolerated, with a 250 µs width at 25 Hz [12].

**Sham procedure**:

For sham-t-VNS, stimulating electrodes were placed on the outer earlobe, which does not contain fibers of the ABVN. A protocol similar to that used for active stimulation was used.[21]


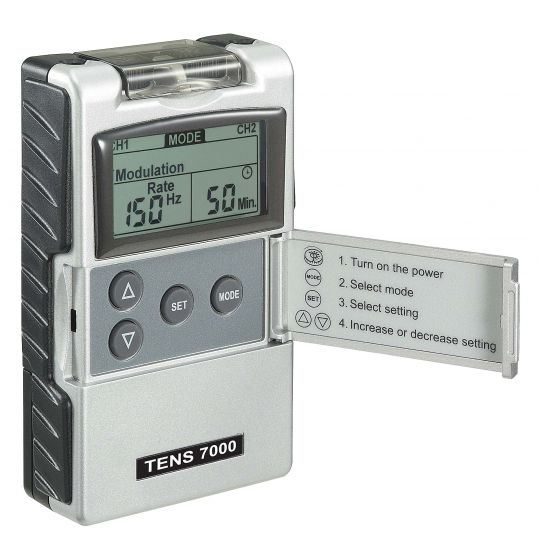


**Figure 1: TENS 7000TM device**


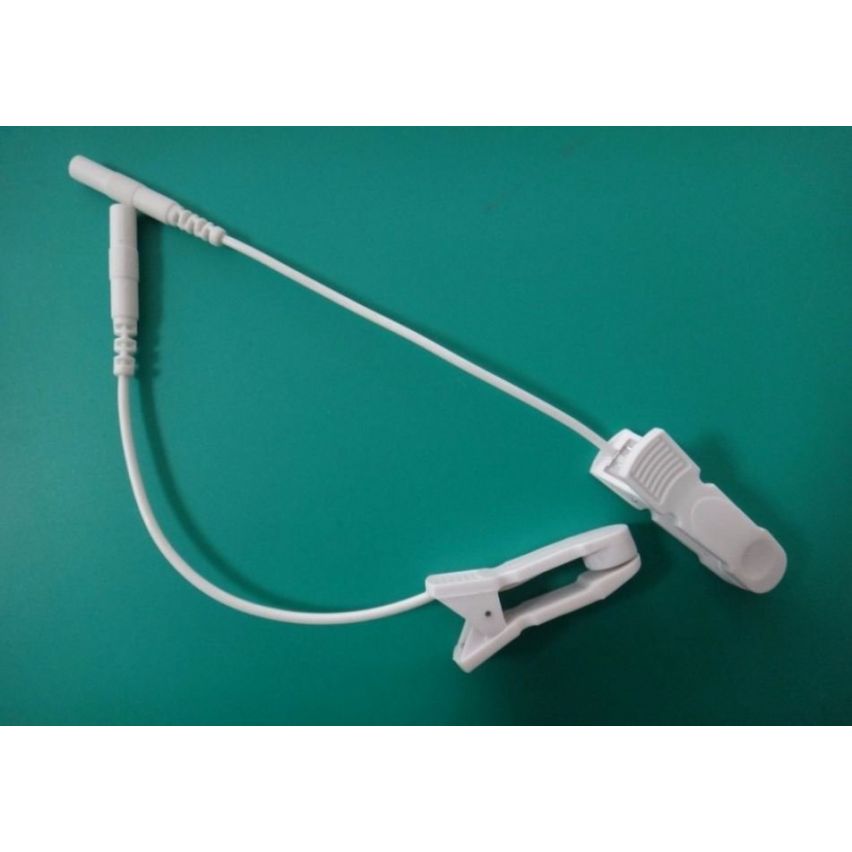


**Figure 2: ear clips used for stimulation of cymba concha**


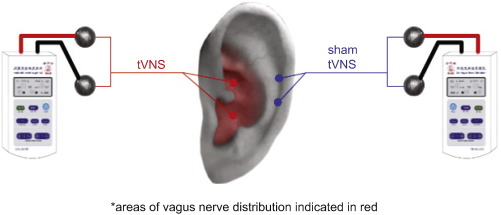


**Figure 3: areas of active tVNS (red) and sham tVNS (blue) stimulation[21]**

All participants were initially evaluated before and after intervention and 4 weeks after the end of the sessions by full medical history, general and musculoskeletal examination, and the following outcome measures:

**OUTCOME MEASURES**

1. Visual analog scale (VAS).
2. Quantitative sensory testing (QST): Pressure pain threshold (PPT).
3. The central sensitization inventory (CSI)
4. Knee injury and Osteoarthritis Outcome Score (KOOS).
5. Physical function tests: Performance-based chair stand test (CST) and timed up and go test.
6. PainDETECT questionnaire.
7. Douleur neuropathique 4 (DN4) questionnaire.
8. Hospital Anxiety Depression Scale (HADS).
9. Kellgren-Lawrence (KL) Radiological classification.
10. **Visual analog scale (VAS):**

On a line measuring one centimeter and graded from 0 to 10, patients were asked to indicate the degree of their knee pain, with "0" denoting no pain and "10" denoting the greatest pain they could imagine. It is also possible to categorize pain as none, mild, moderate, or severe (none = 0, mild = 1-4, moderate = 5-6, and severe = 7-10) [22].

1. **Central sensitization:**
   1. **Pressure pain threshold (PPT):**

Tests of PPT are performed at a diseased site to measure local pain sensitivity as a surrogate for peripheral and/or central sensitization. Tests performed at distant, nonpainful sites test widespread hypersensitivity, a sign of central sensitization [23]. Using standardized instructions, we applied the pressure algometer to the following sites: the OA knee and the contralateral knee (medial joint line) as well as the right elbow over the extensor carpi radialis brevis muscle. We obtained the average of three measurements [24].

The first point at which a pressure feeling turns into a pain sensation was determined using a pressure algometer (Wagner Instruments, Greenwich, USA, PainTestTM FPN 100 Algometer) as shown in figure 4. Using a flat, circular, metal probe with a rubber covering, induced pressure was applied to the locations mentioned before. The algometer was mounted vertically and the pressure was increased. Patients were asked to inform the examiner as soon as they felt any pain. Each measure was followed by a 30-second break. More sensitivity is indicated by lower PPT values [25].

**Figure (4) PainTest™ FPN 100 Algometer**

- 1. **Central Sensitization Inventory (CSI)** [26]**:**

The CSI was created in 2012 as a screening method to determine whether the symptoms being experienced are due to central sensitization or reflect central sensitivity [27]. Together with its initial construct validity, the CSI's psychometric potency and clinical value are satisfactory. There are 25 items in the finished version. CSI values are calculated to a maximum of 100 points and range from 0-4. Subclinical (0–29), mild (30–39), moderate (40–49), severe (50–59), and extreme (60–100) are the five levels [28].

1. **Physical Function Tests**
   1. **Knee injury and Osteoarthritis Outcome Score (KOOS)**[29]**:**

The 42 items that make up the KOOS's five subscales are pain, other symptoms, activities of daily living (ADL), sport and recreation, and knee-related quality of life. Each question receives a score between 0 and 4, which is converted to a score between 0 and 100. More difficulties are indicated by a lower score [29].

- 1. **Timed up and go:**

This test measures how long it takes someone to get up from a regular chair, go three meters away to a line, turn around, and then get back in the chair and seat down. If necessary, the chair's arms can be used as support while standing or sitting. On average, two trials were required [30]. This test and functional mobility have a good association. Healthy older people usually finish the task within just 10 seconds [31].

- 1. **Performance-based Chair Stand Test (CST):**

CST was carried out following the instructions in the Osteoarthritis Initiative manual. The patients were comfortably seated on the floor with their knees flexed little beyond 90 degrees in a chair without armrests. Patients were instructed to rise up five times as soon as they could without using their hands while using a stopwatch. After a countdown from three, time began at "Go" and finished at the fifth stand [32]. The reference value for the CST was 8.50 seconds (95% CI=7.93–9.07 seconds) [33].

1. **PainDETECT** **questionnaire (PD-Q)** [34]**:**

This is a neuropathic pain screening test. Its specificity and sensitivity were first claimed to be 80% and 85%, respectively, for the detection of neuropathic pain caused by back pain. Seven sensory-weighted descriptive questions and two questions describing the temporal and propagation aspects of pain make up this test. Scores between 13 and 18 imply undetermined points; the ultimate score ranges from 0 to 38 likelihood. For neuropathic pain, a score of ≤12 indicates low probability, whereas a score of ≥19 indicates high probability [34].

1. **DN4 questionnaire** [35]**:**

The Douleur Neuropathique 4 questionnaire was created to evaluate neuropathic pain. There are ten questions, and the answers are yes or no. Seven of these items evaluate the intensity of the pain, while the remaining three, depending on the clinical examination, identify the presence of sensory allodynia and touch-needle hypoesthesia [35]. Each item answered as “yes” yields 1 point, and a total score at or above 4/10 is considered positive. This questionnaire has 83% sensitivity and 90% specificity for chronic pain associated with a lesion in the nervous system (central or peripheral) [36].

1. **Hospital Anxiety Depression Scale (HADS)** [37]**:**

The HADS was created to evaluate a patient's level of anxiety and depression as well as the intensity change over time. It includes subscales for depression and anxiety. It consists of 14 different parts in total. Seven of these items (with odd numbers) evaluate anxiety, and the remaining eight (with even numbers) evaluate depression. An overall subscale score of >8 points out of 21 indicates significant anxiety or depressive symptoms [37].

1. **Radiological Imaging:**

The Kellgren-Lawrence (KL) classification was originally described using AP knee radiographs. Each radiograph was given a score between 0 and 4 that corresponded to the degree of OA, with grade 0 denoting the absence of OA and grade 4 denoting severe OA[38].

**Statistical analysis**

Microsoft Excel software was used to code, enter, and analyze the data collected throughout the history for basic clinical examinations and outcome measures. Data was then imported into the Statistical Package for the Social Sciences (SPSS) version 22.0 software system for analysis. We tested the normality of the data using the Shapiro‒Wilk test. Continuous variables were represented by mean and standard deviation (SD), and categorical variables were presented as frequencies and percentages (%). We tested the significance of associations between categorical variables using chi-square or Fisher's exact tests (if >20% of expected values were less than 5). A Mann‒Whitney test was used for continuous data, and a chi-square or Fisher's exact test were used for categorical data, to test for statistical significance between groups. Friedman's test compares the mean ranks between the related groups and indicates how the groups differed. Significance between periods was measured by Dunn's post hoc test. A multiple logistic regression analysis was conducted to assess the relationship between dependent and independent variables. Significant results were defined as a value below 0.05.

**Timeline**

Protocol preparation: 6 months.

Data collection & field work: 2 years.

Data management and statistical analysis: 6 months.

Manuscript writing and finishing: 6 months.

**Ethical Considerations:**

1-The study evaluated Vagal nerve stimulation treatment for patients of osteoarthritis. It is a well-known treatment for other conditions like depression, anxiety, tinnitus, and migraine, with minimal side effects.

2-This form of treatment is a new modality, with several studies supporting its effectiveness and safety, with few reported side effects like hoarseness, dysphonia. So, all patients included in the study received effective and safe treatment without any obligation about the choice of the treatment modalities.

3-An informed written consent in Arabic language was taken from all the participants before taking any data or doing any investigations.

4-This informed consent included full description of the procedure, duration of the study, possible side effects, right of withdrawal from the study at any time, contact details of the investigators and the IRB committee.

5-Patients diagnosed to have knee OA received proper health education and suitable medical treatment whether they are part of the study or not.

**Budget:**

The budget of the study was afforded by the researchers:

| - Preparing protocol | 500 LE |
| --- | --- |
| - Collecting data | 500 LE |
| - Interventions | 7000 LE |
| - Statistical analysis | 1000 LE |
| - Total | 9000 LE |

**Funding**

This research did not receive any specific grant from funding agencies in the public, commercial, or not-for-profit sectors.

**Project management**

G.G.E., N.K.E., M.E.I., N.H.A., and M.A.H. designed the study. G.G.E. collected the clinical data. G.G.E. and M.E.I. analyzed and interpreted the patient data. N.K.E. and M.A.H. interpreted the patient data. All authors discussed the results, contributed to the final manuscript, and approved it. N.K.E. discussed the results and supervised all the research process. All authors approved the final manuscript and agreed to the published version of the manuscript.

**List of abbreviations**

ABVN: Auricular Branch of The Vagus Nerve

ADL: Activity of Daily Living

BMI: body mass index

CSI: Central Sensitization Inventory

CST: Performance-Based Chair Stand Test

DN4: Douleur Neuropathique 4 Questionnaire

HADS: Hospital Anxiety Depression Scale

IL-1β: Interleukin-1 Β

KL: Kellgren-Lawrence Grading System

KOOS: Knee Injury and Osteoarthritis Outcome Score

OA: Osteoarthritis

PPT: Pressure Pain Threshold

QOL: Quality of Life

QST: Quantitative Sensory Testing

RA: Rheumatoid Arthritis

SD: Standard Deviations

TENS: Transcutaneous Electrical Nerve Stimulation

TNF: Tumor Necrosis Factor

tVNS: Transcutaneous Vagus Nerve Stimulation

VAS: Visual Analog Scale

VNS: Vagus Nerve Stimulation

**References**

[1] S. Glyn-Jones *et al.*, “Osteoarthritis,” *The lancet*, vol. 386, no. 9991, pp. 376–387, Jul. 2015, doi: 10.1007/978-3-319-59963-2_9.

[2] F. Berenbaum and Q. J. Meng, “The brain-joint axis in osteoarthritis: Nerves, circadian clocks and beyond,” *Nat. Rev. Rheumatol.*, vol. 12, no. 9, pp. 508–516, 2016, doi: 10.1038/nrrheum.2016.93.

[3] X. Cai, S. Yuan, Y. Zeng, C. Wang, N. Yu, and C. Ding, “New Trends in Pharmacological Treatments for Osteoarthritis,” *Front. Pharmacol.*, vol. 12, p. 701, Apr. 2021, doi: 10.3389/FPHAR.2021.645842/BIBTEX.

[4] J.-P. Pelletier, J. Martel-Pelletier, and S. B. Abramson, “Osteoarthritis, an inflammatory disease: Potential implication for the selection of new therapeutic targets,” *Arthritis Rheum.*, vol. 44, no. 6, pp. 1237–1247, Jun. 2001, doi: 10.1002/1529-0131(200106)44:6<1237::AID-ART214>3.0.CO;2-F.

[5] W. H. Robinson *et al.*, “Low-grade inflammation as a key mediator of the pathogenesis of osteoarthritis,” *Nat. Rev. Rheumatol.*, vol. 12, no. 10, pp. 580–592, Oct. 2016, doi: 10.1038/nrrheum.2016.136.

[6] T. W. O’Neill and D. T. Felson, *Mechanisms of Osteoarthritis (OA) Pain*, vol. 16, no. 5. Current Medicine Group LLC 1, 2018, pp. 611–616. doi: 10.1007/s11914-018-0477-1.

[7] U. Andersson and K. J. Tracey, “Neural reflexes in inflammation and immunity.,” *J. Exp. Med.*, vol. 209, no. 6, pp. 1057–68, Jun. 2012, doi: 10.1084/jem.20120571.

[8] S. E. Rasmussen *et al.*, “Vagal influences in rheumatoid arthritis,” *Scand. J. Rheumatol.*, vol. 47, no. 1, pp. 1–11, Jan. 2018, doi: 10.1080/03009742.2017.1314001.

[9] A. S. Caravaca *et al.*, “A novel flexible cuff-like microelectrode for dual purpose, acute and chronic electrical interfacing with the mouse cervical vagus nerve.,” *J. Neural Eng.*, vol. 14, no. 6, p. 066005, Dec. 2017, doi: 10.1088/1741-2552/aa7a42.

[10] W. Parrish *et al.*, “Modulation of TNF release by choline requires alpha7 subunit nicotinic acetylcholine receptor-mediated signaling.,” *Mol. Med.*, vol. 14, no. 9–10, p. 1, 2008, doi: 10.2119/2008-00079.Parrish.

[11] C. Reardon, K. Murray, and A. E. Lomax, “Neuroimmune Communication in Health and Disease,” *Physiol. Rev.*, vol. 98, no. 4, pp. 2287–2316, Oct. 2018, doi: 10.1152/physrev.00035.2017.

[12] F. A. Koopman *et al.*, “Vagus nerve stimulation inhibits cytokine production and attenuates disease severity in rheumatoid arthritis.,” *Proc. Natl. Acad. Sci. U. S. A.*, vol. 113, no. 29, pp. 8284–9, Jul. 2016, doi: 10.1073/pnas.1605635113.

[13] J. E. Ghia *et al.*, “The Vagus Nerve: A Tonic Inhibitory Influence Associated With Inflammatory Bowel Disease in a Murine Model,” *Gastroenterology*, vol. 131, no. 4, pp. 1122–1130, Oct. 2006, doi: 10.1053/j.gastro.2006.08.016.

[14] E. Ben-Menachem, “Vagus nerve stimulation, side effects, and long-term safety.,” *J. Clin. Neurophysiol. Off. Publ. Am. Electroencephalogr. Soc.*, vol. 18, no. 5, pp. 415–8, Sep. 2001.

[15] B. Bonaz, V. Sinniger, and S. Pellissier, “The vagus nerve in the neuro-immune axis: Implications in the pathology of the gastrointestinal tract,” *Front. Immunol.*, vol. 2, no. 8, p. 1452, Nov. 2017, doi: 10.3389/fimmu.2017.01452.

[16] K. Chakravarthy, H. Chaudhry, K. Williams, and P. J. Christo, “Review of the Uses of Vagal Nerve Stimulation in Chronic Pain Management,” *Curr. Pain Headache Rep.*, vol. 19, no. 12, p. 54, Dec. 2015, doi: 10.1007/s11916-015-0528-6.

[17] D. Schiphof, J. Runhaar, E. J. Waarsing, E. W. van Spil, M. van Middelkoop, and S. M. Bierma-Zeinstra, “The 10-year course of the clinical American college of rheumatology (acr) criteria for hip and knee osteoarthritis in an early symptomatic cohort, data from check,” *Osteoarthritis Cartilage*, vol. 26, pp. S347–S348, Apr. 2018, doi: 10.1016/j.joca.2018.02.691.

[18] H. Kang, “Sample size determination and power analysis using the G*Power software,” *J. Educ. Eval. Health Prof.*, vol. 18, p. 17, Jul. 2021, doi: 10.3352/jeehp.2021.18.17.

[19] H. Yuan and S. D. Silberstein, “Vagus Nerve and Vagus Nerve Stimulation, a Comprehensive Review: Part III,” *Headache J. Head Face Pain*, vol. 56, no. 3, pp. 479–490, Mar. 2016, doi: 10.1111/HEAD.12649.

[20] J. B. Frøkjaer *et al.*, “Modulation of vagal tone enhances gastroduodenal motility and reduces somatic pain sensitivity,” *Neurogastroenterol. Motil.*, vol. 28, no. 4, pp. 592–598, Apr. 2016, doi: 10.1111/nmo.12760.

[21] J. Fang *et al.*, “Early cortical biomarkers of longitudinal transcutaneous vagus nerve stimulation treatment success in depression,” *NeuroImage Clin.*, vol. 14, pp. 105–111, Jan. 2017, doi: 10.1016/J.NICL.2016.12.016.

[22] G. A. Hawker, S. Mian, T. Kendzerska, and M. French, “Measures of adult pain: Visual Analog Scale for Pain (VAS Pain), Numeric Rating Scale for Pain (NRS Pain), McGill Pain Questionnaire (MPQ), Short-Form McGill Pain Questionnaire (SF-MPQ), Chronic Pain Grade Scale (CPGS), Short Form-36 Bodily Pain Scale (SF,” *Arthritis Care Res.*, vol. 63, no. SUPPL. 11, pp. 240–252, Nov. 2011, doi: 10.1002/acr.20543.

[23] C. J. Woolf, “Central sensitization: Implications for the diagnosis and treatment of pain,” *Pain*, vol. 152, no. SUPPL.3. pp. S2–S15, Mar. 2011. doi: 10.1016/j.pain.2010.09.030.

[24] P. Moss, H. A. E. Benson, R. Will, and A. Wright, “Patients With Knee Osteoarthritis Who Score Highly on the PainDETECT Questionnaire Present With Multimodality Hyperalgesia, Increased Pain, and Impaired Physical Function.,” *Clin. J. Pain*, vol. 34, no. 1, pp. 15–21, Jan. 2018, doi: 10.1097/AJP.0000000000000504.

[25] C. Fingleton, K. Smart, N. Moloney, B. M. Fullen, and C. Doody, “Pain sensitization in people with knee osteoarthritis: a systematic review and meta-analysis.,” *Osteoarthritis Cartilage*, vol. 23, no. 7, pp. 1043–56, Jul. 2015, doi: 10.1016/j.joca.2015.02.163.

[26] R. Neblett, “The central sensitization inventory: A user’s manual,” *J. Appl. Biobehav. Res.*, vol. 23, no. 2, p. e12123, Jun. 2018, doi: 10.1111/jabr.12123.

[27] R. Neblett *et al.*, “The Central Sensitization Inventory (CSI): establishing clinically significant values for identifying central sensitivity syndromes in an outpatient chronic pain sample.,” *J. Pain Off. J. Am. Pain Soc.*, vol. 14, no. 5, pp. 438–45, May 2013, doi: 10.1016/j.jpain.2012.11.012.

[28] T. Nishigami, K. Tanaka, A. Mibu, M. Manfuku, S. Yono, and A. Tanabe, “Development and psychometric properties of short form of central sensitization inventory in participants with musculoskeletal pain: A cross-sectional study,” *PLoS ONE*, vol. 13, no. 7, Jul. 2018, doi: 10.1371/JOURNAL.PONE.0200152.

[29] T. Koos, D. Living, and R. Function, “Knee injury and Osteoarthritis Outcome Score,” no. August, pp. 1–5, 2012, doi: 10.1097/00005131-200609001-00018s.

[30] F. Dobson *et al.*, “Reliability and measurement error of the Osteoarthritis Research Society International (OARSI) recommended performance-based tests of physical function in people with hip and knee osteoarthritis,” *Osteoarthritis Cartilage*, vol. 25, no. 11, pp. 1792–1796, Nov. 2017, doi: 10.1016/J.JOCA.2017.06.006.

[31] T. Herman, N. Giladi, and J. M. Hausdorff, “Properties of the ‘Timed Up and Go’ test: More than meets the eye,” *Gerontology*, vol. 57, no. 3, pp. 203–210, 2011, doi: 10.1159/000314963.

[32] E. O. Huber, A. Meichtry, R. A. de Bie, and C. H. Bastiaenen, “Construct validity of change scores of the Chair Stand Test versus Timed Up and Go Test, KOOS questionnaire and the isometric muscle strength test in patients with severe knee osteoarthritis undergoing total knee replacement,” *Man. Ther.*, vol. 21, pp. 262–267, Feb. 2016, doi: 10.1016/j.math.2015.09.012.

[33] T. Nakazono, N. Kamide, and M. Ando, “The Reference Values for the Chair Stand Test in Healthy Japanese Older People: Determination by Meta-analysis.,” *J. Phys. Ther. Sci.*, vol. 26, no. 11, pp. 1729–31, Nov. 2014, doi: 10.1589/jpts.26.1729.

[34] R. Freynhagen, R. Baron, U. Gockel, and T. R. Tölle, “pain DETECT : a new screening questionnaire to identify neuropathic components in patients with back pain,” *Curr. Med. Res. Opin.*, vol. 22, no. 10, pp. 1911–1920, Oct. 2006, doi: 10.1185/030079906X132488.

[35] D. Bouhassira *et al.*, “Comparison of pain syndromes associated with nervous or somatic lesions and development of a new neuropathic pain diagnostic questionnaire (DN4),” *Pain*, vol. 114, no. 1, pp. 29–36, Mar. 2005, doi: 10.1016/j.pain.2004.12.010.

[36] A. Aşkın, A. Özkan, A. Tosun, Ü. S. Demirdal, and F. İsnaç, “Quality of life and functional capacity are adversely affected in osteoarthritis patients with neuropathic pain,” *Kaohsiung J. Med. Sci.*, vol. 33, no. 3, pp. 152–158, Mar. 2017, doi: 10.1016/J.KJMS.2016.12.007.

[37] R. P. Snaith, “The Hospital Anxiety And Depression Scale.,” *Health Qual. Life Outcomes*, vol. 1, p. 29, Aug. 2003, doi: 10.1186/1477-7525-1-29.

[38] B. F. Riecke, R. Christensen, S. Torp-Pedersen, M. Boesen, H. Gudbergsen, and H. Bliddal, “An ultrasound score for knee osteoarthritis: a cross-sectional validation study.,” *Osteoarthritis Cartilage*, vol. 22, no. 10, pp. 1675–91, Oct. 2014, doi: 10.1016/j.joca.2014.06.020.
